# Frailty Screening in Elderly Patients With Hip Fractures: Predicting Poor Outcomes and Optimizing Clinical Management

**DOI:** 10.7759/cureus.88016

**Published:** 2025-07-15

**Authors:** Panagiotis Poulios, Soma Kar, Arijit Mallick

**Affiliations:** 1 Orthopaedics and Trauma, Royal Free Hospital, London, GBR; 2 Geriatrics, Basildon & Thurrock University Hospitals, Mid and South Essex University Hospitals Trust, Essex, GBR; 3 Trauma and Orthopaedics, Basildon & Thurrock University Hospitals, Mid and South Essex University Hospitals Trust, Essex, GBR

**Keywords:** complications, frailty, hip fractures, outcomes, prognosis

## Abstract

Aim: This study aims to determine the prevalence of frailty among older adults with a hip fragility fracture and its correlation with outcomes such as the risk of postoperative complications, mortality, and hospitalization duration.

Patients and methods: The study was a retrospective observational study conducted at a university-affiliated hospital. The study included 99 patients aged over 65 who had been treated for hip fragility fractures. The participants' frailty was assessed using the frail-to-fit index calculator, and Glegg's criteria were used to stratify the patients into four severity levels. The study employed univariate and multivariate analyses to investigate the correlation between frailty severity and outcomes such as the risk of complications, mortality, and length of hospital stay, as well as to examine the relationship between frailty and other factors.

Results: The study found that 61% of the patients were classified as frail. Among the participants, 37.4% developed at least one postoperative complication, and 11.1% died. Patients with lower frailty scores had a lower risk of developing complications and mortality compared to those with advanced frailty. The hospitalization duration was significantly longer for patients with high frailty scores than those with low scores.

Conclusion: This study provides important insights into managing older adults with hip fragility fractures and highlights the importance of addressing frailty in clinical practice. The study concluded that frailty is a common phenomenon among older adults with hip fragility fractures and is associated with a poorer prognosis, including an increased risk of postoperative complications, mortality, and prolonged hospitalization. Therefore, assessing frailty is essential for improving clinical outcomes when planning a patient's care and discussing their prognosis.

## Introduction

Due to demographic change, there is an increasing proportion of older adults in our society. As the population ages, the number of proximal femur fractures is expected to increase significantly, leading to a greater cause of hospitalization and socioeconomic burden [1]. Hip fractures serve as a marker condition to understand the challenges of caring for older people in modern healthcare services. By 2050, it is projected that there will be six million cases of hip fractures worldwide [2]. Moreover, hip fractures are associated with over £1 billion per year in health and social services. This single injury incurs a total cost equivalent to approximately 1% of the entire National Health Service (NHS) budget [3]. These figures provide awareness of how hip fractures are perceived globally and pose a serious concern at the individual and population levels. The elderly population faces numerous, complex challenges, including compromised physiology, cognitive decline, and multiple comorbidities [3-5]. Therefore, they should receive appropriate, multidisciplinary care tailored to their specific needs.

Frailty is an umbrella term encompassing the physiological, social, and psychological parameters that create the vulnerability phenotype due to decreased reserves in response to stressors [6,7]. It is essential to recognize that frailty is a dynamic and progressive entity directly linked with the cumulative decline of multiple organ systems [8]. It is not part of the normal aging spectrum [9-11]. Another concerning feature of frailty is that it is an independent risk factor for adverse outcomes [12,13]. Recognizing and assessing the frail phenotype is a matter of utmost importance. Among the essential tools in the clinician's armamentarium is the electronic Frailty Index (eFI) [6]. Risk stratification, particularly estimating mortality risk, is vital in medical decision-making and optimal management of older patients.

The optimal care and management of frail seniors necessitate a robust and efficient framework that incorporates a multidisciplinary and holistic approach, assisted by an array of screening and decision-making tools that pragmatically assess and stratify risks [14,15]. It is not yet clear and quantifiable which risk factors significantly correlate with the patient's adverse outcomes. Recognizing the most impactful variables can be a helpful tool for patient and population screening, aiding clinical judgment through timely and evidence-based medicine management methods [16].

The primary objective of this study is to determine whether the frailty score can serve as a reliable predictor of outcomes in elderly patients who have suffered hip fractures. The study hypothesizes that the frailty score is a significant predictor of poor outcomes, including longer hospital stays, postoperative complications, higher readmission rates, and increased mortality rates within 30 days after surgery.

## Materials and methods

Study design and participants

This retrospective cohort study was conducted over eight weeks, from January 2018 to February 2018, at Basildon & Thurrock University Hospital. The study focused on patients with hip fractures who underwent surgical management as part of the hospital's frailty pathway, which provided coordinated care from orthopedic and geriatric specialists per NICE guidelines. The hospital was chosen for its comprehensive geriatric services and adherence to best practices in managing elderly patients with hip fractures. During this period, 115 patients were admitted with hip fractures. However, five patients under 65 years old were excluded to focus on the elderly population, which is typically more affected by hip fractures.

Additionally, eight patients with periprosthetic fractures were excluded due to differing treatment protocols, three patients were excluded as they were either unfit for surgery due to terminal conditions or unable to provide consent, and patients with incomplete data were also excluded. After applying these exclusion criteria, 99 patients were eligible for study participation. Ethical approval was obtained from the relevant institutional review board. Table 1 provides a detailed overview of the demographic and clinical characteristics of the study participants.

**Table 1:** Clinical characteristics of the study participants SD: Standard Deviation; AMTS: Abbreviated Mental Testing Score; ASA: American Society of Anaesthesiologists Physical Status Classification System

Clinical characteristic	All patients (n=99)
Age, mean ± SD	82 ± 4
Female sex	61.9%
Residence before admission (%)	
Own house	77.1%
Residential care home	15.7%
Nursing care home	7.2%
Pre-fracture mobility (%)	
Freely mobile without aids	38.1%
Mobile outdoors with one aid	19.6%
Mobile outdoors with two aids or frame	25.8%
Some indoor mobility but never goes outdoors without help	14.4%
No functional mobility	2.1%
Low AMTS preoperatively (0-3)	3.1%
High Postoperative Delirium score (AMT4)	29.4%
Nutritional status (%) - Mini Nutritional Assessment (MNA)	
Normal	35.4%
At risk for malnutrition	37.5%
Malnourished	27.1%
No comorbidities (%)	1.8%
High ASA grade (>3) (%)	14.6 %

Calculation of the frailty score

The frailty score was calculated using the electronic Frailty Index (eFI) tool. It is a validated tool used to identify and grade frailty in older adults using routinely collected data from electronic health records (EHRs) [17]. It is based on the cumulative deficit model of frailty, which suggests that an individual with a greater number of health deficits is at an increased risk of frailty.

$eFI\,score = Number\,of\,deficits\,present/36$

Calculation Steps

The eFI uses a list of 36 deficits, including clinical signs, symptoms, diseases, disabilities, and abnormal test results. Each deficit is scored as 1 if present and 0 if absent for an individual patient. The total number of deficits present is summed. The eFI score is calculated by dividing the number of deficits present by the total number of possible deficits (36).

For example, if a patient has nine deficits, the eFI score would be 9/36 = 0.25.

Deficits Included

The 36 deficits cover a range of health problems, such as symptoms and abnormal laboratory values (e.g., dizziness, memory issues, visual impairment), diseases (e.g., respiratory disease, hypertension), and disabilities (e.g., mobility or activity limitations).

The eFI is automatically calculated in EHR systems using coded patient data, with no additional clinical assessment required. The resulting scores were further stratified into four groups, as depicted in Table 2.

**Table 2:** Frailty grades

Electronic Frailty Index	Frailty Grades
0 – 0.12	No frailty
>0.12 – 0.24	Mild Frailty
>0.24 – 0.36	Moderate Frailty
>0.36	Severe Frailty

Primary and secondary outcomes

The primary objective of the study was to determine the risk of developing postoperative complications during inpatient care, such as acute kidney injury (AKI), lower respiratory tract infection (LRTI), postoperative anemia, urinary tract infection (UTI), and death. The study aimed to correlate the risk with various proposed determinants, such as age, sex, frailty score, preoperative Abbreviated Mental Test (AMT) score, nutritional status assessed by the Subjective Global Assessment (SGA) tool, pre-injury residence, and pre-injury mobility. The study's secondary objective was to examine the correlation between the number of hospitalization days and the abovementioned variables.

Statistical analysis

Statistical analyses were conducted using SPSS 5 (Released 1993; SPSS Inc. Chicago), for Windows to assess changes in the analyzed variables over the observation period. For continuous parametric data, independent t-tests and one-way analysis of variance (ANOVA) were employed to compare means, assuming normal distribution and homogeneity of variance. Mann-Whitney and Kruskal-Wallis tests were used to analyze non-normally distributed continuous data. Categorical data were analyzed using Fisher's exact test and χ² tests. A p-value of less than 0.05 was considered statistically significant.

To enhance clarity and transparency, we report sample sizes for each analysis and provide measures of central tendency and variability (means with standard deviations for parametric data and medians with interquartile ranges for non-parametric data). Confidence intervals were calculated to quantify the precision of estimates where applicable. The study's limitations, including potential biases and the use of an older software version, are acknowledged.

## Results

One hundred and fifteen patients were admitted with hip fractures during the study. Five patients were excluded due to age <65 years, and eight for periprosthetic fracture. Most patients admitted to our program had surgery for their hip fracture to avoid the risks of recumbency; three patients with hip fractures were unfit for surgery and at the end of life. In conclusion, ninety-nine patients were eligible for study participation.

**Figure 1:**
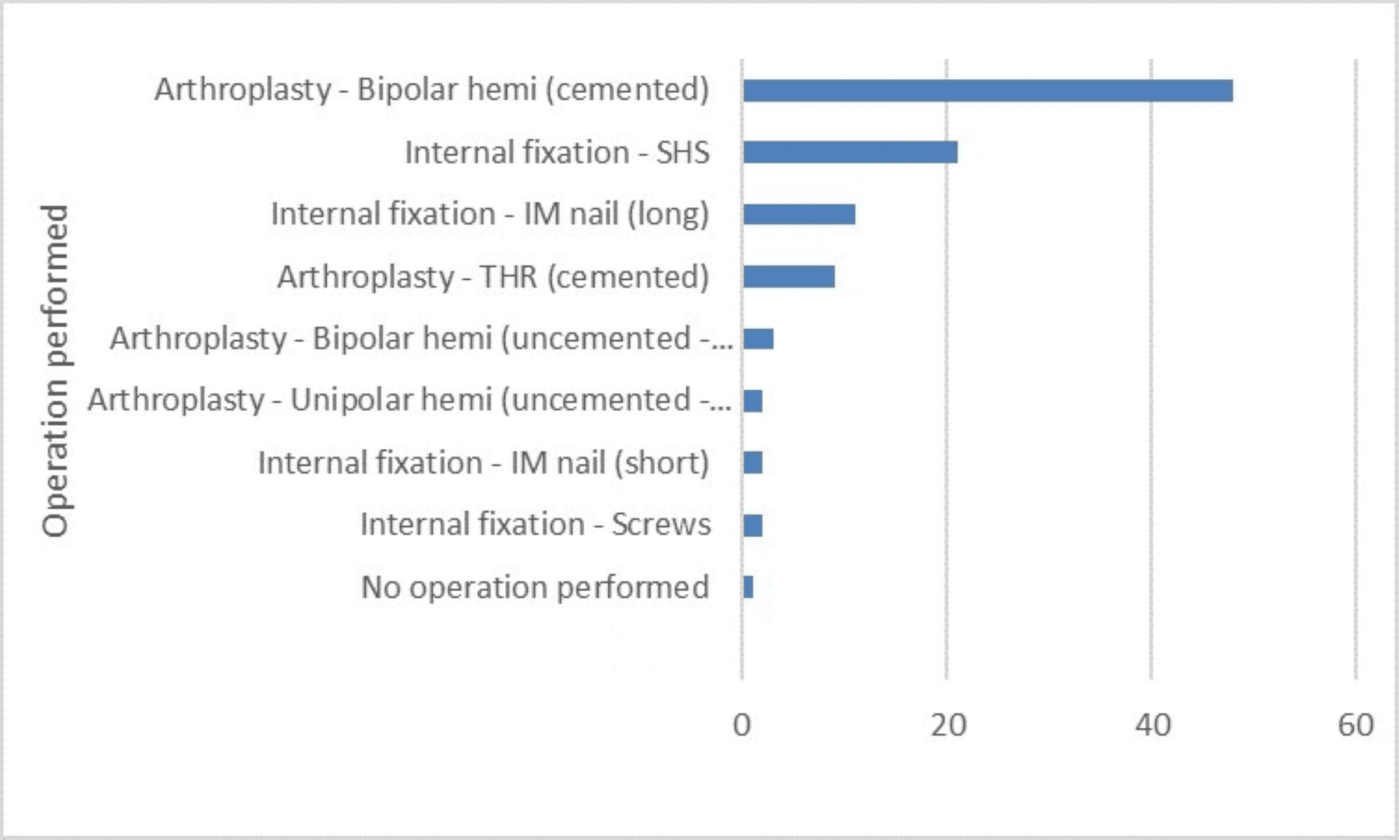
Overview of the operations performed for the hip fractures SHS: Sliding Hip Screw, IM: Intramedullary nail, THR: Total Hip Replacement

Sixty-one (51%) participants were classified as frail based on the Frailty score index. The majority of the participants (61.9 %) were female and lived in the community before hip fracture (77.1%), with reasonable mobility (38.1%). A substantial portion of the participants were at risk of malnutrition (37.5%) and malnourished (27.1%). The most common surgical intervention was cemented hemiarthroplasty (48%) (Figure 1).

In 37.6% of cases, surgery was conducted within 36 hours of admission. The remaining cases encountered delays due to medical reasons, such as the necessity for coagulation reversal or medical review, and logistical reasons, such as the availability of theatre lists. During admission, 3.1% of patients received a low AMTS assessment. 

The most prevalent postoperative complications were AKI and LRTI, which occurred in 7.92% of cases. Other complications included postoperative anemia and UTI. The most frequently observed frailty score ranged from 0.07 to 0.14, indicating mild frailty, followed by moderate frailty.

**Figure 2:**
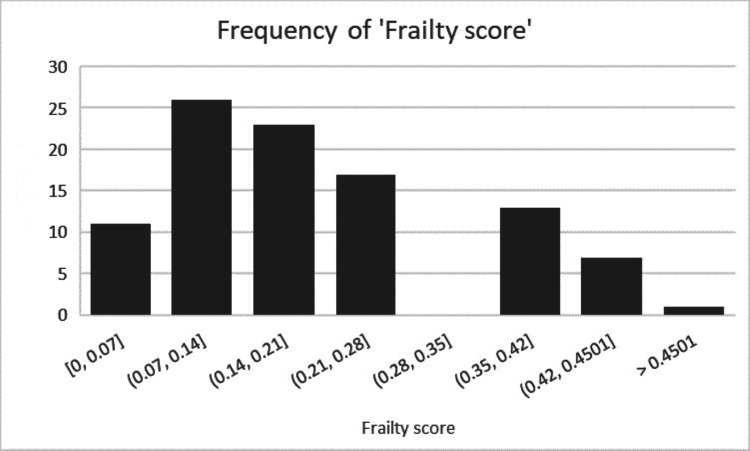
Distribution of the frailty score; the Y axis represents the number of patients

The most prevalent frailty score was between 0.07 and 0.14, representing mild frailty followed by moderate frailty, as illustrated in Figure 2.

Postoperative complication analysis

**Figure 3:**
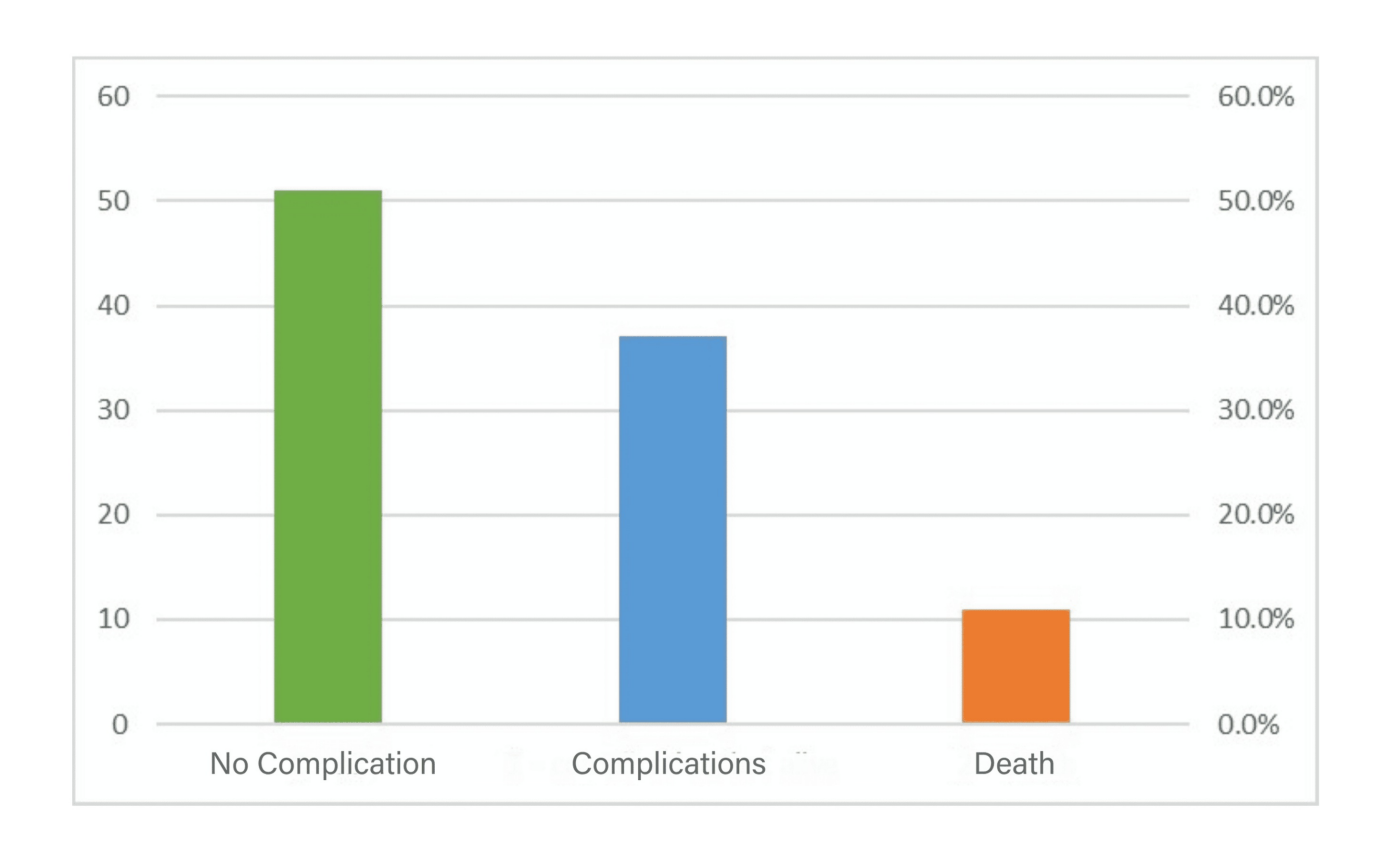
Distribution of the complications in the patient population The green column represents the percentage of no complication, the blue column the percentage of cases with complications, and the orange column the percentage of deaths

To facilitate the analysis, we created three subcategories regarding postoperative complications: No complication, at least one complication, and death. The results are illustrated in Figure 3.

**Figure 4:**
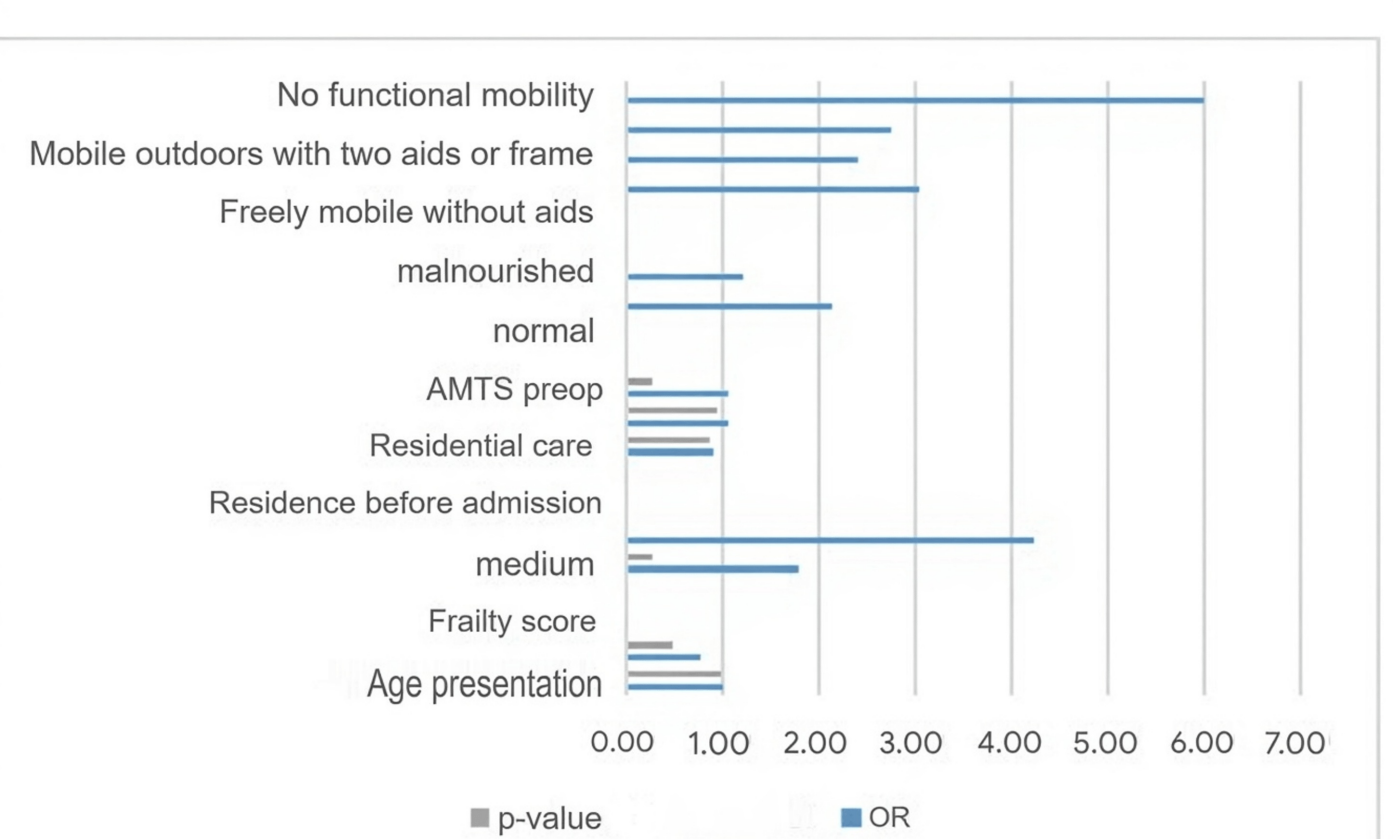
Histogram of the univariate analysis: factors influencing postoperative outcomes AMTS: Abbreviated Mental Test Score

We attempt to interpret the results using the agreed-upon group of factors (age, sex, frailty score, preoperative AMT score, nutritional status, pre-injury residence, and pre-injury mobility) and the association of the response with each covariate separately. The results of the analysis are represented in Figure 4.

From the above analysis, only the frailty score is significantly associated with the postoperative complication variables. Then, we endeavor to perform multivariate analysis to examine relationships between multiple risk factors and quantify them. This analysis includes only patients with complete data, n=80. The results are represented in Table 3.

**Table 3:** Complications by frailty status OR: Odds Ratio; 95% CI: 95% Confidence Interval

Effects	OR	95% CI	P-value	
Frailty Score (medium VS low)	1.74	0.57 - 5.34	0.3315	
Frailty Score (high VS low)	5.1	1.64 - 15.85	0.005	

We then attempt to visualize the results of the above model by predicting the probability of falling into a specific postoperative complication category (nil, complication, death) for each frailty score level (low, medium, high). From the analysis, we obtained the following results, illustrated in Table 4.

**Table 4:** Probability of postoperative complications based on frailty scores

	Probability of Post-Op Complication		
Frailty Score	Nil Complications	Complications	Death
Low	59.7%	32.8%	7.4%
Medium	46.0%	41.7%	12.3%
High	22.6%	48.4%	29.0%

**Figure 5:**
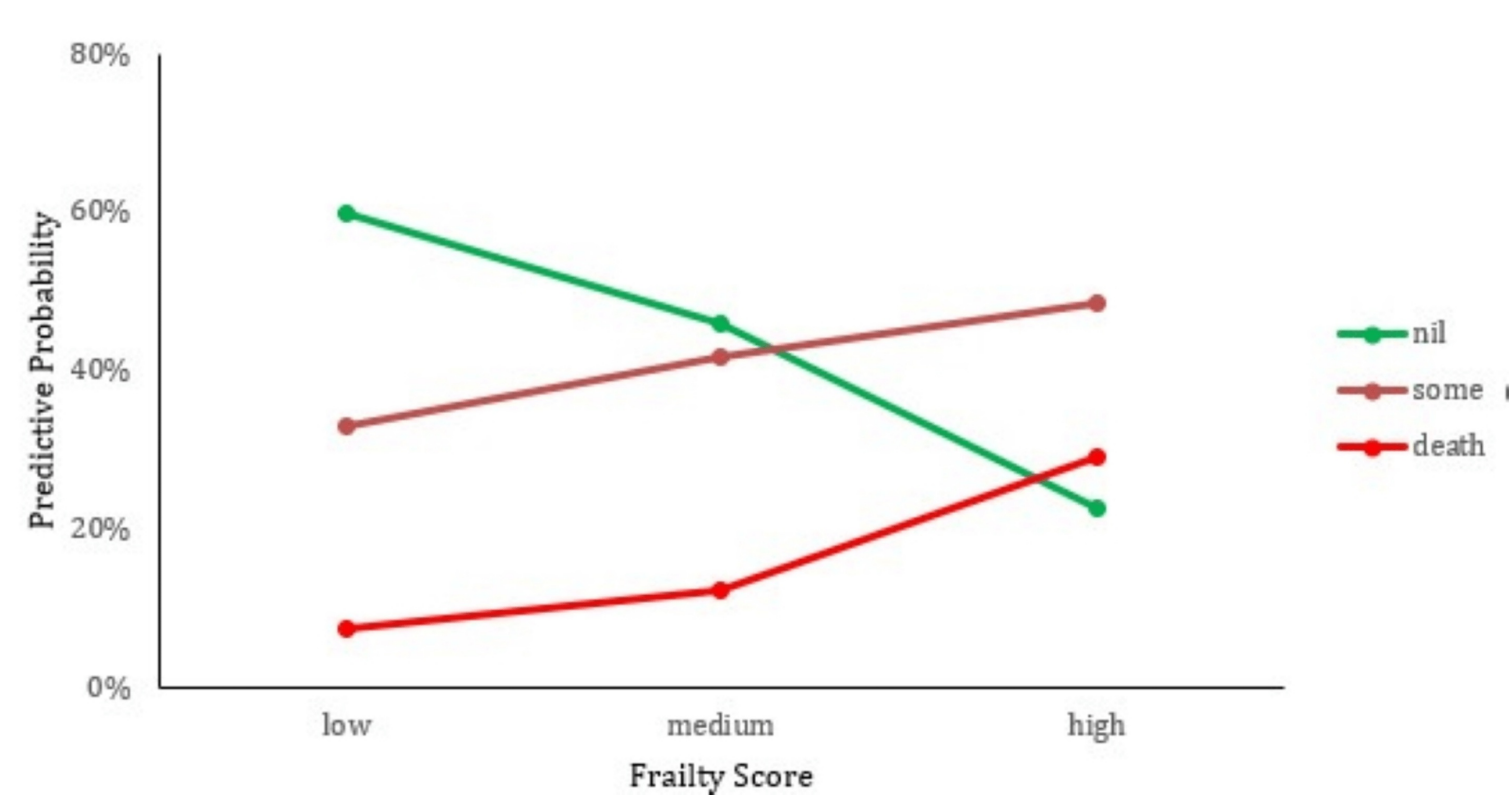
Association of the degree of frailty score and the risk of postoperative complications

We interpret the results using the established group of factors (age, sex, frailty score, pre-op AMT score, nutritional status, pre-injury residence, and mobility) and the association of the response with each covariate separately with the univariate analysis method (Figure 5).

From the above analysis, only the frailty score is significantly associated with the postoperative complication variable. There is a clear association between higher frailty scores and increased risk of severe postoperative outcomes, including death. Patients with medium or high frailty scores are more likely to suffer postoperative complications or even death than those with low frailty scores.

Hospitalization day analysis

The number of days in the hospital is calculated as the difference between the date of admission to A&E (Accident & Emergency) and the date of discharge from the ward. Key results include a mean of 14.3 days, a median of 11.9 days, an interquartile range (IQR) of 10.4 days, a standard deviation (SD) of 9.4 days, and a range of 2.2 to 61.1 days.

**Figure 6:**
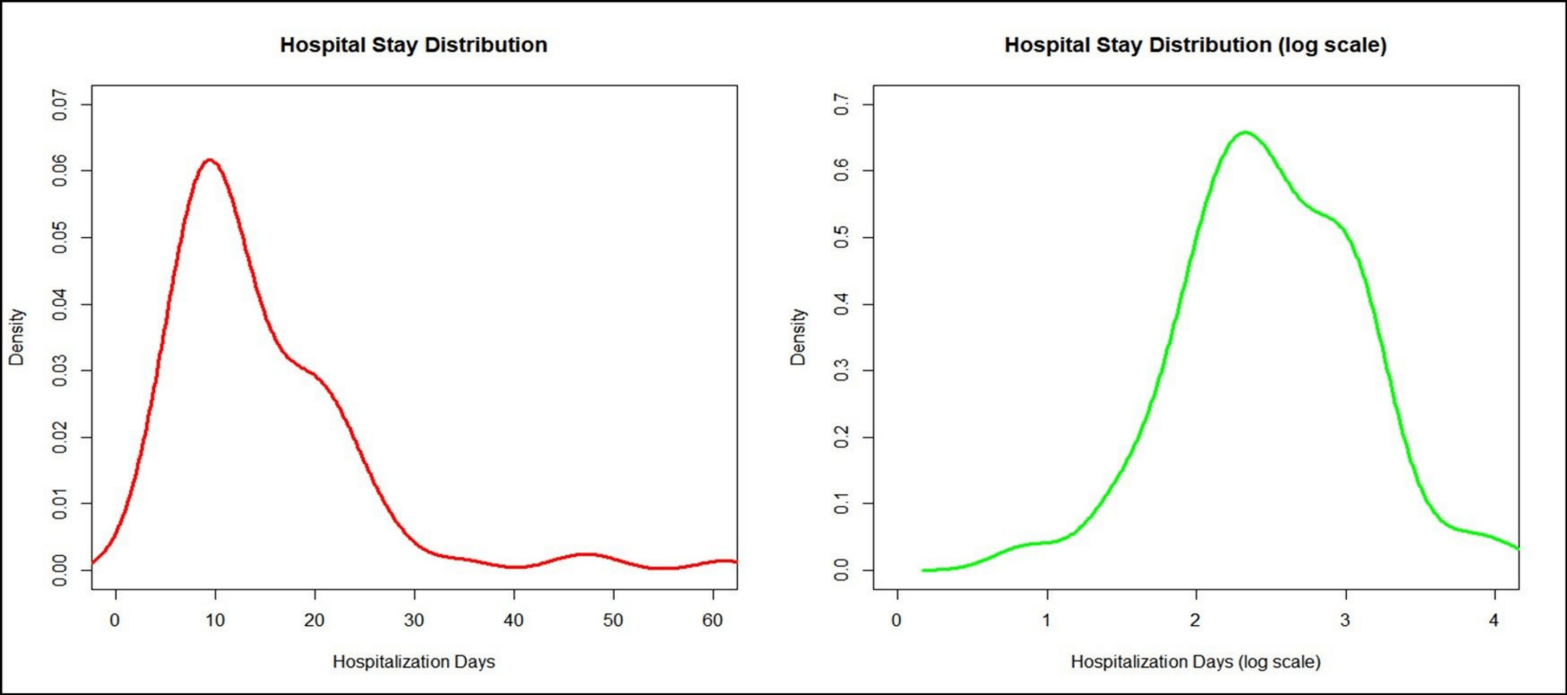
Days of hospitalization distribution Left Graph: Original data form; Right Graph: Logarithmic transformation of the data

The original data (graph left) follows a non-parametric, negatively skewed distribution. To improve the validity of the statistical analysis, we proceed with a logarithmic transformation of the data (right graph) (Figure 6).

**Figure 7:**
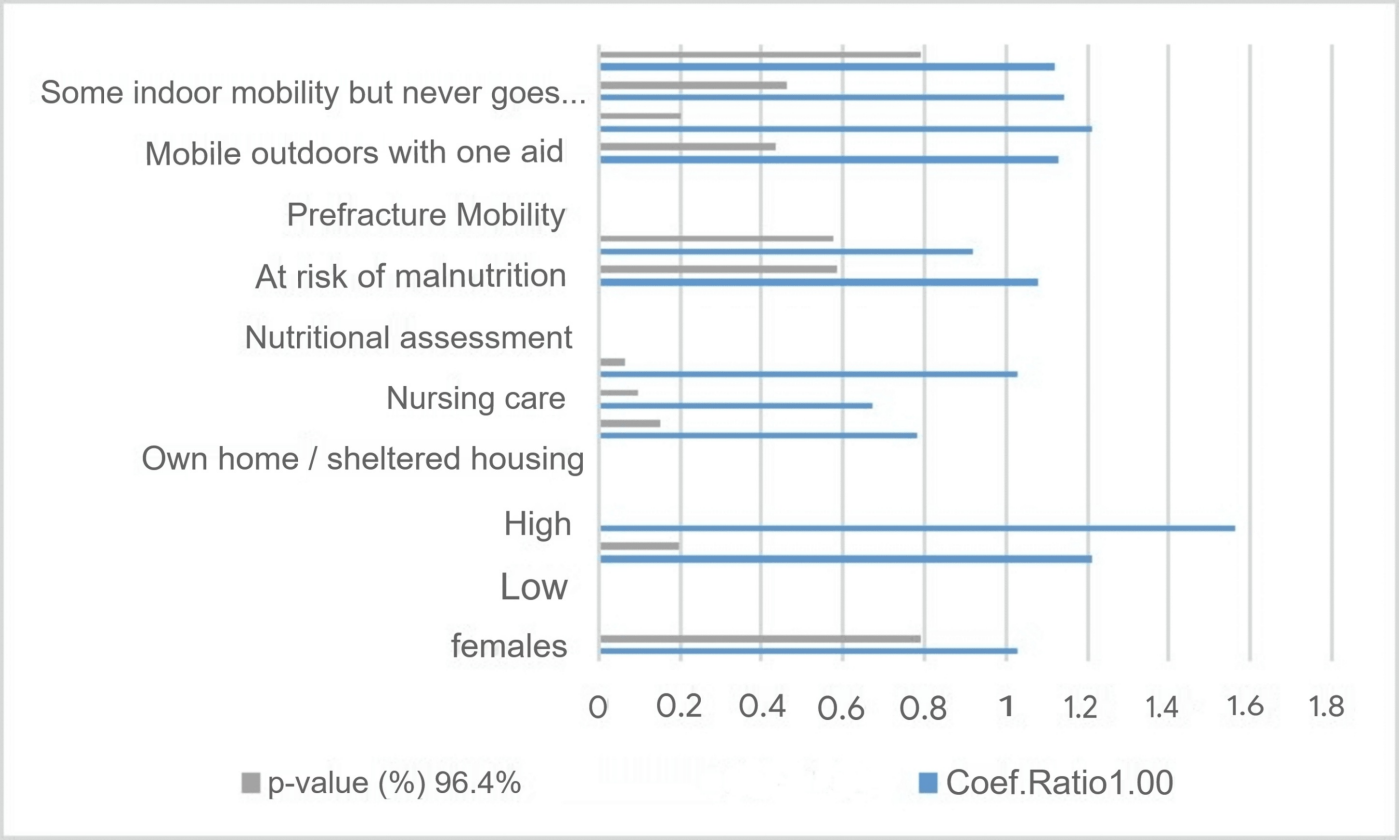
Histogram of univariate analysis

To interpret the results, we used a set of factors (age, sex, frailty score, preoperative AMT score, nutritional status, pre-injury residence, and pre-injury mobility) and the UVA method to determine how the response correlated with each covariate separately (Figure 7). The figure suggests that mobility, nutritional status, care settings, gender, and socioeconomic factors have a significant influence on the study's outcome. The strength of these associations varies across variables, as indicated by their coefficient ratios and confidence intervals.

We use only patients with complete data, n=81 patients, which we include in the regression analysis. The inference drawn is that patients with a high frailty score have a significantly higher risk of prolonged hospitalization (Coef. Ratio: 1.55, 95% CI: 1.44-2.10, p-value: 0.5%), while the medium frailty score does not show a statistically significant correlation (p-value: 13.3%) (Table 5).

**Table 5:** Average predicted hospital stay (days) by the frailty score Coef. Ratio: Coefficient ratio; 95% CI: 95% Confidence interval

Effect	Coef. Ratio	95% CI	p-value
Frailty Score (Medium vs low)	1.26	0.93 - 1.71	13.3%
Frailty Score (High vs low)	1.55	1.44 - 2.10	0.5%

Patients with medium frailty scores stayed 26% longer in the hospital after surgery than those with low frailty scores, but the effect is insignificant (P=13.3%). Patients with high frailty scores stayed 55% longer in the hospital, as compared to patients with low frailty scores, with the effect being significant (p=0.5%)

**Figure 8:**
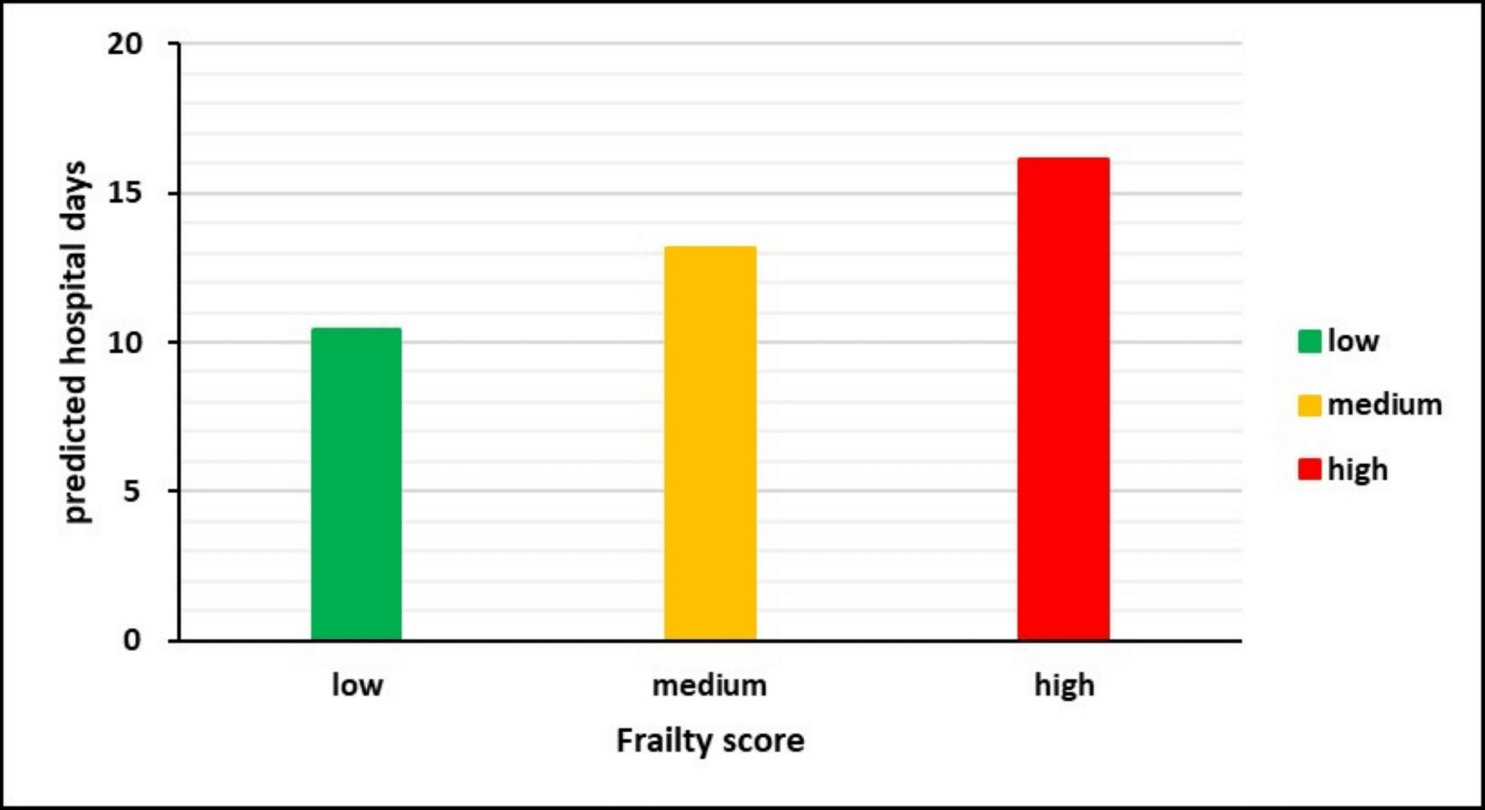
Predicted hospital stay duration based on frailty scores

Next, we attempted to visualize the results of the above model by predicting the expected hospital stay for each Frailty Score level (low, medium, high). Results are depicted in Table 6 and Figure 8. Patients with medium frailty scores are expected to stay three days longer in the hospital after surgery compared to patients with low frailty scores.

**Table 6:** Correlation between the severity of the frailty with the days of hospital stay

Frailty Score	Predicted hospital stay (days)
Low	10.4
Medium	13.1
High	16.1

## Discussion

Frailty has been recognized as a significant factor that contributes to poor outcomes in older adults with hip fractures. Several studies have investigated the relationship between frailty and outcomes in this patient population.

A systematic review and meta-analysis published in 2019 analyzed 22 studies, including over 18,000 patients with hip fractures. The study found that frailty was associated with an increased risk of mortality, functional decline, and complications such as delirium, infections, and pressure ulcers in this patient population [18].

Another study published in 2020 analyzed data from over 1,300 older adults with hip fractures and found that frailty was a significant predictor of postoperative complications, functional decline, and mortality [19]. The study also found that frailty was more strongly associated with adverse outcomes than age or comorbidities.

A study published in 2022 investigated the impact of frailty on outcomes in older adults with hip fractures in a rehabilitation setting. The study found that frailty was associated with more extended hospital stays, higher rates of readmissions, and poorer functional outcomes [20].

Overall, previous research has consistently shown that frailty is a crucial factor in predicting poor outcomes in older adults with hip fractures. Identifying frailty can help healthcare providers optimize clinical management to improve outcomes and reduce healthcare costs.

The main research question of this study is to determine whether frailty scores can predict outcomes in elderly patients with hip fractures. The study claims that the frailty score is a significant predictor for poor outcomes, including extended hospital stays, postoperative complications, readmission rates, and mortality rates within 30 days of surgery. The study concludes that identifying frailty in elderly patients with hip fractures is crucial for predicting outcomes and optimizing clinical management. Incorporating frailty screening into routine clinical practice can help healthcare providers accurately assess patient risk and make more informed decisions about interventions to improve patient outcomes.

This research builds upon the existing literature on the importance of frailty screening in predicting outcomes for elderly patients with hip fractures. Previous studies have shown that frailty predicts adverse outcomes, including increased morbidity and mortality rates, extended hospital stays, and decreased functional outcomes. However, this study contributes to the literature by highlighting the relationship between frailty scores and poor outcomes in elderly patients with hip fractures. The findings of this study provide valuable insights into the role of frailty screening in predicting outcomes for this patient population, which can inform clinical practice and optimize care.

The results of this study suggest that the frailty score is a crucial factor in predicting poor outcomes in elderly patients with hip fractures. The findings have significant implications for clinicians and healthcare systems, as identifying frailty allows for better risk assessment and management of elderly patients with hip fractures. Frailty screening should be routine in clinical practice to identify patients at risk for complications and adverse outcomes.

To optimize patient outcomes, healthcare providers should consider preoperative interventions, such as nutrition and exercise, to enhance patient strength and resilience. Postoperative interventions, such as monitoring for complications and ensuring appropriate pain management, can also help reduce the risk of adverse outcomes and improve recovery times.

It is important to acknowledge the limitations of this study. Firstly, the study was conducted at a single center, which may limit the generalizability of the results to the broader population. Additionally, the study focused solely on patients who underwent surgery for hip fractures, meaning the findings may not apply to those who did not undergo surgery. 

Furthermore, this study did not include a formal a priori power calculation, as all eligible patients admitted during the study period were included in the analysis. The sample size of 99 was based on the available population over eight weeks at this single center. Although significant associations were found for some outcomes, we cannot rule out the possibility of a type II error (the failure to detect actual effects) for smaller effect sizes or subgroup analyses. 

Finally, other factors, such as comorbidities and functional status, may also impact the prediction of outcomes in this patient population.

Therefore, further research is necessary to explore the generalizability of these findings across different healthcare settings and patient populations. Additionally, future studies should investigate the role of preoperative and postoperative interventions in improving outcomes for frail elderly patients with hip fractures and examine the impact of incorporating frailty screening into routine clinical practice for elderly patients with hip fractures.

Overall, this study highlights the importance of frailty screening in predicting outcomes for elderly patients with hip fractures. By identifying frailty in these patients, healthcare providers can better assess risk and optimize clinical management to improve outcomes and reduce healthcare costs. Further research is necessary to fully understand the implications of frailty screening and interventions for this patient population.

## Conclusions

This study highlights the significant role of frailty screening in predicting outcomes for elderly patients with hip fractures. The findings suggest that higher frailty scores are associated with increased risk of postoperative complications, extended hospital stays, higher re-admission rates, and increased mortality rates within 30 days of surgery. As a result, identifying frailty in these patients is crucial for healthcare providers to optimize clinical management and improve patient outcomes. Incorporating frailty screening into routine clinical practice can aid healthcare providers in accurately assessing patient risk and making more informed decisions about interventions to improve patient outcomes. The clinical implications of these findings are clear, and they can guide healthcare providers in their approach to managing frail elderly patients with hip fractures. Overall, this study provides valuable insights into the importance of frailty screening in predicting outcomes for this patient population, which can inform clinical practice and optimize care.
